# Performance assessment of medical service for organ transplant department based on diagnosis-related groups: A programme incorporating ischemia-free liver transplantation in China

**DOI:** 10.3389/fpubh.2023.1092182

**Published:** 2023-04-06

**Authors:** Jianjun Lu, Zhuochen Lin, Ying Xiong, Hui Pang, Ye Zhang, Ziyi Xin, Yuelin Li, Zhiqing Shen, Wei Chen, Wujun Zhang

**Affiliations:** ^1^Department of Quality Control and Evaluation, The First Affiliated Hospital, Sun Yat-sen University, Guangzhou, China; ^2^Center for Information Technology and Statistics, The First Affiliated Hospital, Sun Yat-sen University, Guangzhou, China; ^3^Department of Pancreato-Biliary Surgery, The First Affiliated Hospital, Sun Yat-sen University, Guangzhou, China; ^4^Center of Hepato-Pancreato-Biliary Surgery, The First Affiliated Hospital, Sun Yat-sen University, Guangzhou, China

**Keywords:** diagnosis-related groups, medical services, liver transplant, performance evaluation, ischemia-free liver transplant

## Abstract

**Background:**

In July 2017, the first affiliated hospital of Sun Yat-sen university carried out the world’s first case of ischemia-free liver transplantation (IFLT). This study aimed to evaluate the performance of medical services pre- and post-IFLT implementation in the organ transplant department of this hospital based on diagnosis-related groups, so as to provide a data basis for the clinical practice of the organ transplant specialty.

**Methods:**

The first pages of medical records of inpatients in the organ transplant department from 2016 to 2019 were collected. The China version Diagnosis-related groups (DRGs) were used as a risk adjustment tool to compare the income structure, service availability, service efficiency and service safety of the organ transplant department between the pre- and post-IFLT implementation periods.

**Results:**

Income structure of the organ transplant department was more optimized in the post-IFLT period compared with that in the pre-IFLT period. Medical service performance parameters of the organ transplant department in the post-IFLT period were better than those in the pre-IFLT period. Specifically, case mix index values were 2.65 and 2.89 in the pre- and post-IFLT periods, respectively (*p* = 0.173). Proportions of organ transplantation cases were 14.16 and 18.27%, respectively (*p* < 0.001). Compared with that in the pre-IFLT period, the average postoperative hospital stay of liver transplants decreased by 11.40% (30.17 vs. 26.73 days, *p* = 0.006), and the average postoperative hospital stay of renal transplants decreased by 7.61% (25.23 vs.23.31 days, *p* = 0.092). Cost efficiency index decreased significantly compared with that in the pre-IFLT period (*p* < 0.001), while time efficiency index fluctuated around 0.83 in the pre- and post-IFLT periods (*p* = 0.725). Moreover, the average postoperative hospital stay of IFLT cases was significantly shorter than that of conventional liver transplant cases (*p* = 0.001).

**Conclusion:**

The application of IFLT technology could contribute to improving the medical service performance of the organ transplant department. Meanwhile, the DRGs tool may help transplant departments to coordinate the future delivery planning of medical service.

## Introduction

Liver transplant (LT) is the only effective treatment for patients with end-stage liver disease. However, there are many difficulties in its clinical application. On the one hand, there are many patients in the world who cannot receive transplants due to a shortage of donor organs. For example, the growing demand for liver grafts remains an unsolved challenge for the transplant community (1). According to the report of the Organ Procurement and Transplantation Network (OPTN), the number of new liver transplantation waiting list registrations in the United States (12,767 cases) continued to grow in 2019 (2). Donor organ shortage is common in various countries to varying degrees, including the persistent shortage of deceased donor organs for transplantation and the limited number of potential donors after brain death (3). On the other hand, ischemia during an organ transplant is a temporary interruption of blood flow to the organ during the transplant. This can occur during the organ’s removal from the donor and transport to the recipient (known as cold ischemia), and during the surgical implantation process (known as warm ischemia). Ischemia can lead to tissue damage and inflammation, leading to poor transplant outcomes. In liver transplantation, prolonged cold ischemia is associated with an increased risk of graft dysfunction and primary non-function, while warm ischemia during implantation can lead to delayed graft function and reduced graft survival.

Previous studies suggested that longer cold/warm ischemia time was identified as an independent risk factor for moderate to severe ischemia–reperfusion injury (4). Longer cold ischemia is associated with poorer liver transplantation outcomes (5). A similar situation has been reported in kidney transplantation. For example, the current gold standard for preserving donor kidneys is static refrigeration at 4°C. However, this can lead to renal ischemia–reperfusion injury, which adversely affects graft survival and function (6, 7). In fact, risk factors associated with primary graft nonfunction include donor age, cold ischemia time, warm ischemia time, and graft quality. A study based on data from the United Kingdom Transplant Registry suggests that cold ischemia duration of more than 8 h is a risk factor for primary non-function and reduced graft survival after liver transplantation (8). Moreover, currently available donor organs are more likely to come from marginal donors (such as those from older, obese individuals), and these marginal organs tend to have a higher risk of ischemia–reperfusion injury. Therefore, how to limit or even reverse graft ischemia is of great significance for improving the success rate of transplantation (9).

Hypothermic preservation is the storage of organs or tissues in a relatively low temperature environment (2 ~ 8°C), often used to preserve the liver, kidney, heart, etc. Currently, the majority of deceased donor livers are preserved *via* the cold static preservation technique in transport containers with melting ice as the medium for maintaining that temperature. Donor livers usually need to be stored in cold storage for a long period of time during transport from donor to recipient hospital prior to transplantation. Cold ischemia time was defined as the time interval between liver acquisition and liver reperfusion. In the clinical practice of organ transplantation, the cold/warm ischemia process of donor organ after circulatory death may affect the viability of donor organ.

Currently, many measures are used to reduce ischemic damage to candidate organs in order to optimize donor organ quality. For example, normothermic regional perfusion and *ex situ* perfusion techniques can enhance the preservation, evaluation, resuscitation, and/or repair of damaged organs, thereby improving overall organ quality (3). Use of hypothermic machine perfusion immediately before liver transplantation reduces the chance of perfusion dysfunction during early transplantation. Hypothermic machine perfusion can also reduce the risk of delayed graft function and improve graft survival (10).

In July 2017, the first affiliated hospital of Sun Yat-sen university successfully carried out the first ischemia-free liver transplantation (IFLT) in the world. As ischemia-free organ transplant technology has significantly improved the performance of medical services in the field of liver transplant, a professional team from the organ transplantation department of this hospital was awarded the Grand Prize in the 2020 international quality innovation competition. At present, ischemia-free transplant is considered as a milestone in the history of organ transplant, which could be extended to the transplantation fields of heart, lung and kidney, and has a broad application prospect.

Scientific evaluation of medical service performance is helpful to improve the clinical practice of organ transplant. Diagnosis-related groups (DRGs) are cases combination tools developed by American scholars in the 1970s, which are suitable for the evaluation of short-term hospitalization medical service performance and medical insurance payment (11, 12). Now DRGs were widely used in many countries and regions in North America, Europe, Oceania and Asia (13–15). Previous studies have used DRGs tool to evaluate the service performance and hospitalization cost of care for cancers (16, 17), spinal surgery (18, 19) and carotid stent implantation (20). However, to our knowledge, there have been no studies using the DRGs tool to evaluate the performance of medical care for organ transplant. Therefore, this study aimed to evaluate and compare the overall medical service performance of the organ transplant department between the pre-IFLT and post-IFLT periods.

## Methods

### Data source

In 2012, medical institutions in Guangdong began to use a universal discharge summary to record the information of hospitalized patients. This summary, known as the First Page of Medical Records (FPMR), contains basic information (demographics, admission date, discharge dates, etc.), diagnostic information (primary diagnosis, other diagnoses, treatment outcomes, etc.), surgical procedure information (primary operation, other operations, etc.), and medical cost information. The 10th International Classification of Diseases (ICD-10) and the ninth International Classification of Diseases (ICD-9-CM-3) were used to code diagnosis and surgical procedures, respectively. The medical service analysis system of hospitalized patients with DRGs provided by the software company (Wuhan Dongfang Succe Software Co., LTD.) was used for data analysis.

In this study, we retrospectively analyzed the FPMR of patients admitted to the organ transplant department at the hospital studied from 2016 to 2019. The inclusion criteria of this study were: (a) date of discharge from hospital between January 2016 and December 2019; (b) The discharged department was organ transplantation department. The exclusion criteria was that key information about the case (diagnosis, surgery, medical costs, etc.) was missing. Based on the above criteria, 9,718 records were collected for further analysis. Meanwhile, since organ re-transplantation cases were not grouped separately in the DRGs system, re-transplantation cases (including liver transplantation and kidney transplantation cases) were not excluded from the study and were included as separate cases. There were 0, 1, 1 and 2 cases of liver re-transplantation, while there were 3, 5, 7 and 8 cases of kidney re-transplantation in 2016, 2017, 2018 and 2019, respectively.

Ischemia of the donor organ during the acquisition and transplantation stage leads to ischemia–reperfusion injury. In July 2017, the first affiliated hospital of Sun Yat-sen university was the first worldwide to carry out ischemia-free liver transplantation. This non-ischemic organ transplantation technology pioneered by the first affiliated hospital of Sun Yat-sen university worldwide is a new method that continuously applies local perfusion at normal temperature during the stages of donor organ acquisition, preservation and transplantation to ensure continuous blood supply to organs (Supplementary Figure S1). In this study, the period from January 1, 2016 to June 30, 2017 was defined as the “pre-IFLT period” and the period from July 1, 2017 to December 31, 2019 was defined as the “post-IFLT period.” Specifically, 3,510 hospitalizations were included in the analysis during the pre-IFLT period, including 497 (14.16%) solid organ recipients, and 6,208 hospitalizations were included in the analysis during the post-IFLT period, including 1,134 (18.27%) solid organ recipients. In addition, there were 54 IFLT transplant recipients in the post-IFLT period.

### DRGs selection methods

**Figure 1:**
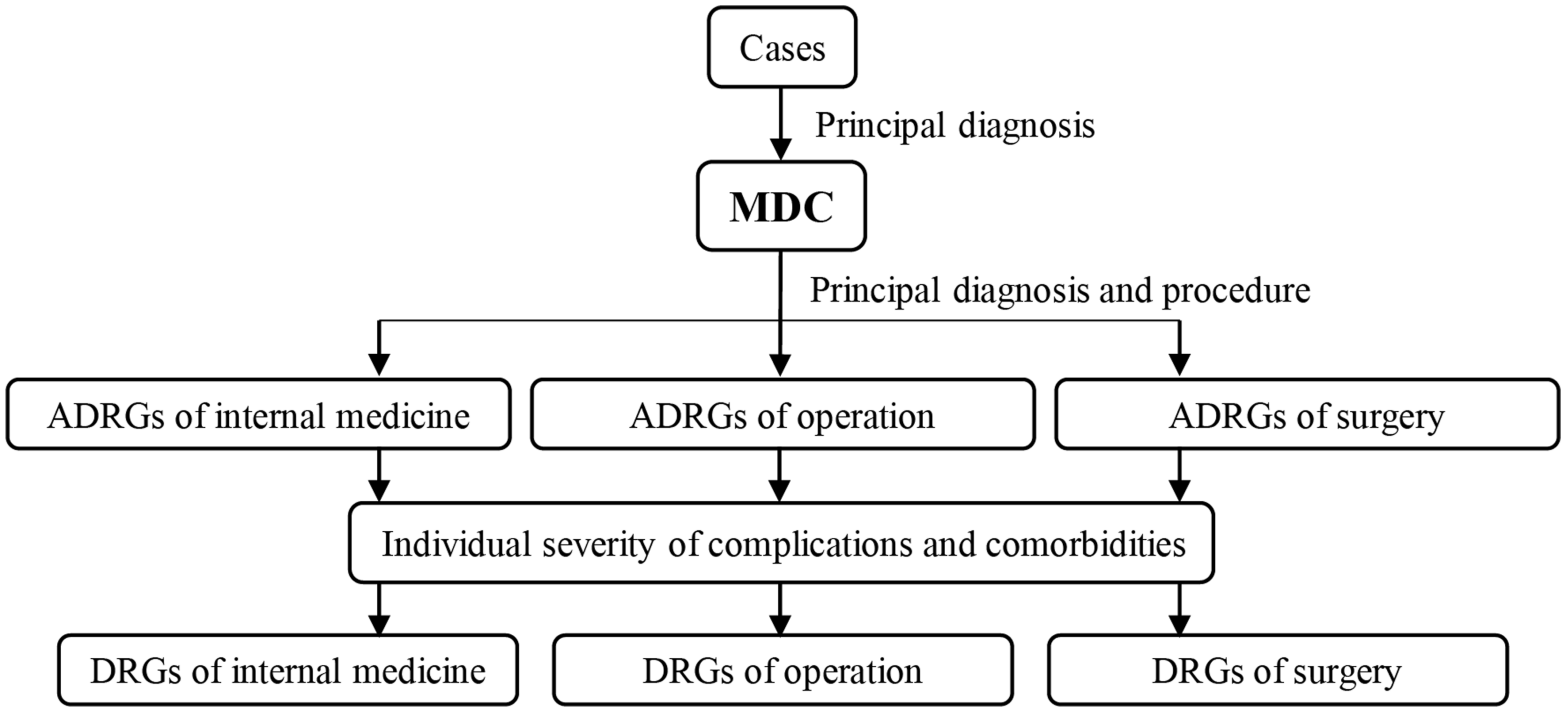
CN-DRGs grouping path. MDC, Major Diagnostic Category. CN-DRGs, China diagnosis-related groups.

Beijing Institute of Hospital Management pioneered DRGs researches in China. In 2011, Beijing DRGs grouping scheme began to be promoted and applied in China. In 2015, the former National Health and Family Planning Commission launched the 2014 version of the China DRGs (CN-DRGs) grouping scheme applicable to the coded data environment of diseases and surgeries in China based on the Beijing DRG grouping scheme. Subsequently, the National Health Commission of China released a revised version of the 2014 edition: the CN-DRGs Grouping Scheme 2018 edition. As a Guangdong Province specific DRG has not yet been developed, the CN DRG (2018 edition) was used as a risk adjustment tool in this study. CN DRGs were divided into 26 main diagnostic categories (MDC) and 806 DRGs (21) according to the characteristics of the cases (age, sex, diagnosis, operation, comorbidities and complications, etc.). Figure 1 shows the group path of the CN DRG.

#### DRG evaluation indicators

Previous studies suggest that DRGs-based evaluation can help improve the comparability of cases and the reliability of evaluation results. It is an important part of medical performance evaluation in medical service research and can provide a basis for rational decision-making (22–24). According to the evaluation methods of DRGs, we used six objective indicators of medical service performance to evaluate the available scope, efficiency and safety of medical services for the organ transplant specialty. The average levels of DRGs indices of medical institutions in Guangdong Province were used as the standards in the calculation of DRG index data (Table 1).

**Table 1:** Health system performance evaluation indicators based on DRGs.

Dimension	Indicators	Evaluation contents
**Availability**	Number of DRGs	The range of services available
	Total weight	Total output of in-patient services
	Case-mix index (CML)	Average technical difficulty level of treating diseases in each discipline
**Efficiency**	Charge efficiency index (CEI)	Cost of treating similar diseases
	Time efficiency index (TEI)	Time for treating similar diseases
**Safety**	Inpatient mortality of low-risk group cases (IMLRG)	Mortality of diseases that are extremely unlikely to cause death

#### Service availability indicators

The number of DRGs, total weight, and case mix index (CMI) were used to assess the availability of medical service to reflect the scope of care, total output and the adjusted technical difficulty of case treatment. The total weight and CMI are calculated as follows:


$$\begin{array}{l} \mathrm{Total weight}=\sum \mathrm{Each}\;\mathrm{DRG}\;\mathrm{weight}\times \\ \;\;\;\;\;\;\;\;\;\;\;\;\;\;\;\;\;\;\;\;\;\;\;\;\;\;\;\;\;\;\;\;\;\;\;\;\mathrm{Number of cases in each}\;\mathrm{DRG}\; \end{array}$$

$$\mathrm{CMI}=\frac{\mathrm{Total weight}}{\mathrm{Number of cases in Guangdong}}$$

By dividing the average cost of each DRG group by the average cost of all cases in Guangdong Province, each DRG weight was calculated. DRG weights were averaged to create CMI.

Besides, relative weight (RW) is analyzed in this study. In the DRGs evaluation system, the RW is the weight given to each DRG according to its degree of resource consumption, reflecting the degree of hospital resource consumption of the DRGs relative to other diseases. The higher the value, the higher the resource consumption of the case portfolio. The formula is: RW = average cost of this DRGs/average cost of all disease groups. Therefore, the RW indicator was used in this study as a measure of severity and resource utilization in a particular DRG group.

#### Service efficiency indicators


$$\begin{array}{l} Costratio\left(k^{c}\right)=\\ \;\;\;\;\;\;\;\frac{averagecostof\;a\;DRG\;inGuangdongProvince\;\left(c_{i}\right)}{averagecostof\;a\;DRG\;in\;CN-DRGs\;\left({\bar{C}}_{i}\right)} \end{array}$$

$$\begin{array}{l} Averagelengthofstay\;\left(ALOS\right)\;ratio\;\left(k^{l}\right)=\\ \;\;\;\;\;\;\;\;\;\;\;\;\;\;\;\;\;\;\frac{averagelengthof\;a\;DRG\;inGuangdongProvince}{averagelengthof\;a\;DRG\;in\;CN-DRG\left({\bar{L}}_{i}\right)} \end{array}$$

The Cost efficiency index (CEI) and the Time efficiency index (TEI) were used to evaluate the efficiency of medical services. The CEI and TEI are the results of individual medical institutions compared to the average of all hospitals included in the CN DRG assessment in terms of medical costs and length of stay (LOS). Therefore, the higher the CEI and TEI values, the lower the efficiency of medical services. TEI and CEI greater than 1, respectively, indicate that the time efficiency and cost efficiency required to treat the same diseases are lower than the standard samples (22). CEI and TEI are calculated as follows:


$$\mathrm{CEI}=\frac{\sum_{j}k_{j}^{c}n_{j}}{\sum_{j}n_{j}}$$

$$\mathrm{TEI}=\frac{\sum_{j}k_{j}^{l}n_{j}}{\sum_{j}n_{j}}$$

where n_j_ represents the number of cases in DRGj. The weighted averages of k^c^ and k^l^ represent TEI and CEI.

#### Medical safety indicators

Inpatient mortality of low-risk group cases (IMLRP) is the mortality cases from a disease that is highly unlikely to cause death and can therefore be used to reflect the safety of medical services (25). IMLRP is calculated as follows: (a) Calculation of in-hospital mortality rates (Mi) for each DRG, (b) Calculate the logarithm of Mi (Ln (Mi)), (c) Calculate the mean and standard deviation of L (Mi), and (d) Calculation of a mortality risk score. A mortality risk score of 1 was defined as a low-risk group.

In the DRGs evaluation system, the low-risk group of cases refers to the low risk of death cases generated by the DRGs grouping in a certain year. Specifically, the low-risk group is the DRG group in which the mortality rate of cases is less than minus one standard deviation. In DRG evaluation system, low-risk group mortality is often used to measure the safety of medical services. The basic principle is that once a non-critical case of death occurs, it means that the cause of death is likely to lie not in the disease itself but in the clinical or managerial process.

### Statistical analysis

Comparing independent samples was done using Mann–Whitney U tests with continuous variables expressed as average values. Chi-square tests or Fisher exact tests are used to compare categorical variables expressed as counts or percentages (%). Statistical significance was judged based on *p* < 0.05 values (bilaterally). The time trend of continuous variables can be represented by line charts. The analyses were conducted using SPSS version 22.0 (IBM Corp., Armonk, NY, United States).

## Results

### Sample characteristics

In this study, we collected the medical records of 9,718 inpatients admitted to the organ transplant department of this hospital between January 2016 and December 2019. Of those, 3,510 were hospitalized during the pre-IFLT period and 6,208 were hospitalized during the post-IFLT period. The demographic characteristics of liver and renal transplant recipients in the pre- and post-IFLT periods were analyzed, respectively. As shown in Table 2, there was no significant difference in the age and gender distribution of liver transplant patients before and after IFLT. Meanwhile, the proportion of kidney transplant patients older than 40 years increased after IFLT compared with those before IFLT (*p* = 0.027), with no significant difference in gender distribution.

**Table 2:** Demographic characteristics of organ transplant recipients in the pre- and post-IFLT periods.

Demographic characteristics		Pre-IFLT	Post-IFLT	*χ*^2^ value	*p* value
		*n* (%)	*n* (%)		
*LT*					
Age	<40	41 (20.92)	75 (20.83)	0.001	0.981
	> = 40	155 (79.08)	285 (79.17)		
Gender	Female	29 (14.80)	49 (13.61)	0.148	0.701
	Male	167 (85.20)	311 (86.39)		
*RT*					
Age	<40	186 (63.05)	424 (55.57)	4.877	0.027
	> = 40	109 (36.95)	339 (44.43)		
Gender	Female	89 (30.17)	267 (34.99)	2.217	0.136
	Male	206 (69.83)	496 (65.01)		

LT, liver transplant; RT, renal transplant; IFLT, ischemia-free liver transplant.

### Comparison of clinical income structure

Compared with the pre-IFLT period, the income structure during the post-IFLT period was significantly optimized (*p* < 0.001). Specifically, although the average hospitalization cost increased by 11.44% during the post-IFLT period, the proportion of drug costs decreased by 17.65%. In addition, the proportion of material expense, examination expense, service expense and treatment income increased by 46.62, 4.14, 21.62 and 51.64%, respectively, (Table 3).

**Table 3:** Comparison of clinical income structure of the organ transplant department between the pre- and post-IFLT periods.

Income structure	Pre-IFLT	Post-IFLT	Percentage change (%)	*χ*^2^ value	*p* value
Average cost (Yuan)	28780	32073	11.44	—	—
Drug cost (%)	47.42	39.05	−17.65	938.783	<0.001
Material cost (%)	7.55	11.07	46.62		
Examination cost (%)	20.99	21.86	4.14		
Service income (%)	6.29	7.65	21.62		
Treatment income (%)	9.78	14.83	51.64		
Other costs (%)	7.97	5.53	−30.61		

IFLT, ischemia-free liver transplant.

### Application of the ischemia-free transplant technique

**Figure 2:**
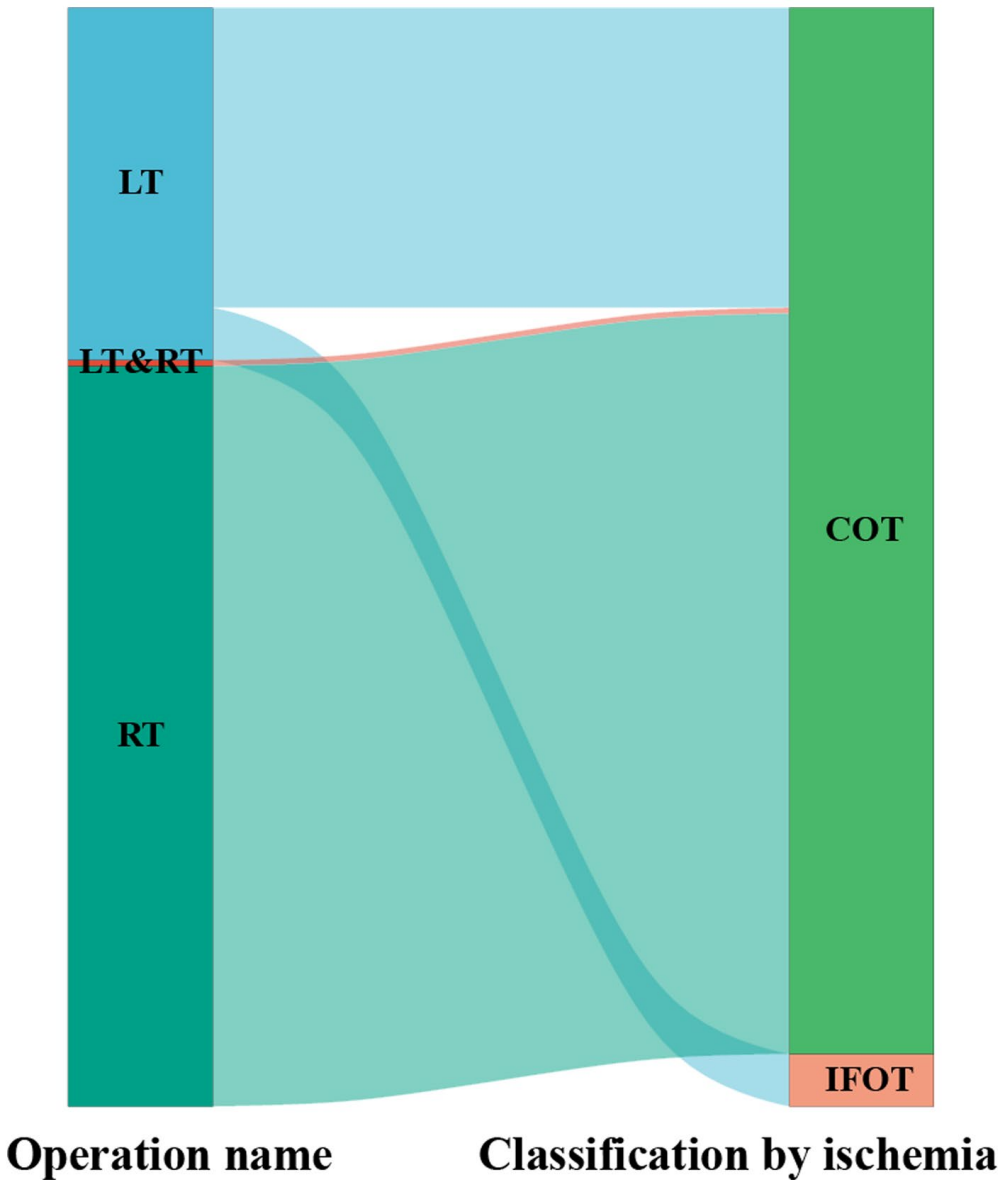
Sankey diagram depicting the selection of surgical methods in a tertiary hospital from July 2017 to December 2019. LT, liver transplant. RT, renal transplant. COT, conventional organ transplant. IFOT, ischemia-free organ transplant.

The Sankey chart was used to visualize the application of ischemia-free transplantation technique in the organ transplant department during the post-IFLT period (Figure 2). A total of 1,132 organ transplants were performed, including 363 liver transplants, 763 kidney transplants, 6 combined liver and kidney transplants and 2 other organ transplants. Specifically, of the 363 liver transplants in the post-ILFT period, 54 were IFLTs and 309 were conventional liver transplants (CLT).

### Promotion of clinical service capacity by the new technique

**Figure 3:**
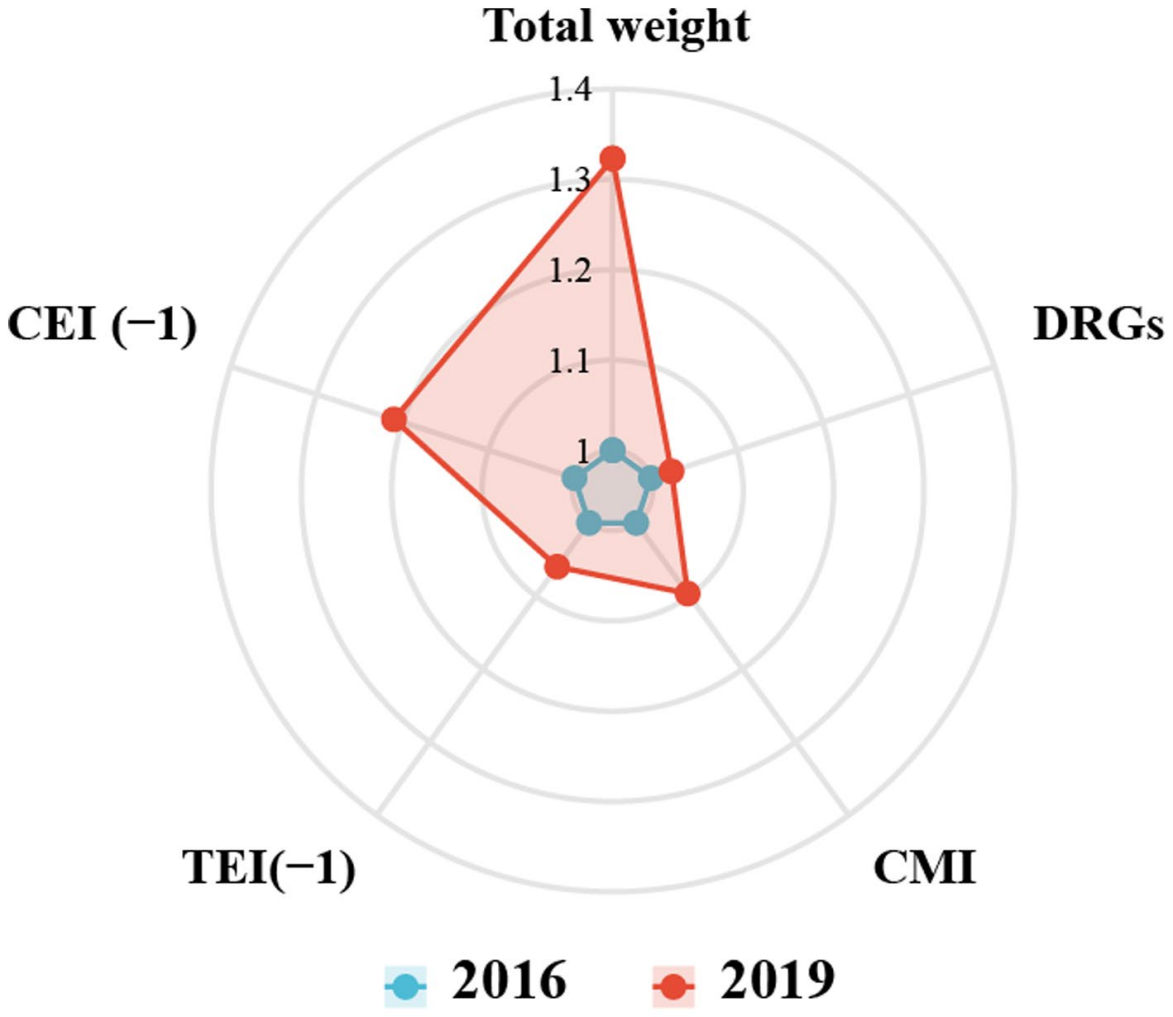
Comparison the medical service performance for department of organ transplant between 2016 and 2019. DRGs, diagnosis-related groups. CMI, Case Mix Index. TMI, time efficiency index. CEI, charge efficiency index.

As shown in Figure 3, the clinical service capability parameters of the organ transplantation department of this hospital in the post-IFLT period were better than those in the pre-IFLT period, suggesting that the implementation of ischemia-free liver transplantation improved the clinical service capability of this specialty in the hospital where this study was conducted.

### Availability of medical services

#### DRGs, CMI and RW comparison

**Figure 4:**
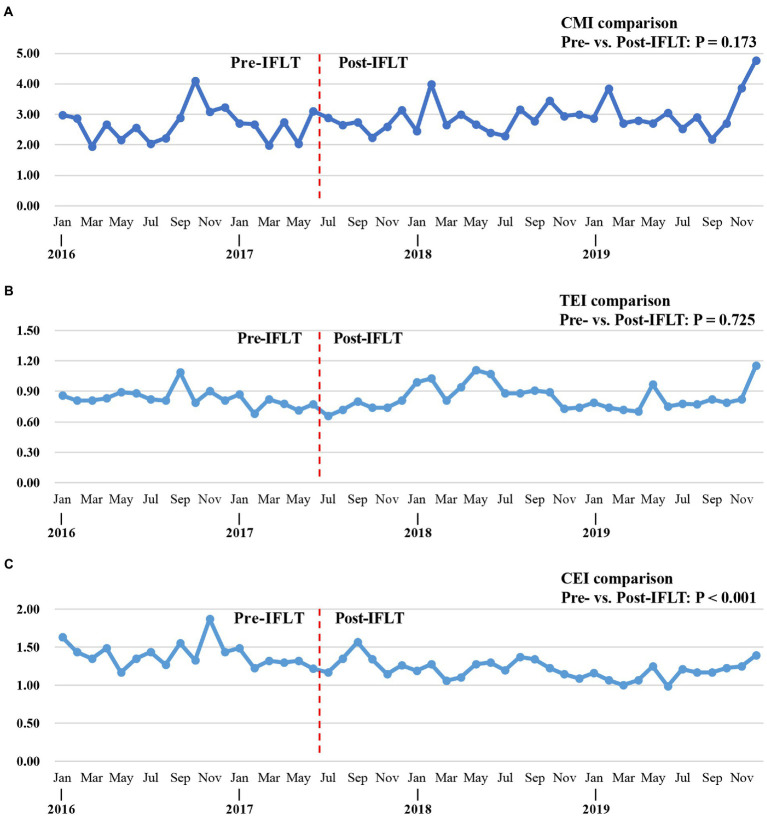
Trend of point estimates of DRGs indicators from 2016 to 2019. **(A)** Trend of point estimates for CMI (pre- vs. post-IFLT, *p* = 0.173). **(B)** Trend of point estimates for TEI (pre- vs. post-IFLT, *p* = 0.0.725). **(C)** Trend of point estimates for CEI (pre- vs. post-IFLT, *p* < 0.001). DRGs, diagnosis-related groups. CMI, Case Mix Index. TMI, time efficiency index. CEI, charge efficiency index.

As shown in Table 4, the number of DRGs in the organ transplantation department during the pre-IFLT period and post-IFLT period was 149 and 188, respectively. The CMI value in pre-IFLT period and post-IFLT period was 2.65 and 2.89, respectively. There was no significant difference in CMI between the two periods (*p* = 0.173, Figure 4A). The distribution of RW values in the post-IFLT period changed significantly compared with that in the pre-IFLT period (*p* < 0.001). In the post-IFLT period, the proportion of RW 5–10 cases increased by 3.81 (8.38% vs. 12.19%), while the proportion of RW 1.5–5 cases decreased by 2.42% (10.91% vs. 8.49%). The proportion of cases with RW < 1.5 and RW > 10 showed only slight changes. In addition, there were no low-risk deaths in either period.

**Table 4:** Evaluation of clinical competence of the organ transplant department in the pre- and post-IFLT periods by DRGs.

Clinical competence evaluation	Pre-IFLT	Post-IFLT	*χ*^2^/*Z* value	*p* value
*Diagnosis and treatment ability*				
Discharge patients	3510	6208	—	—
DRGs	149	188	—	—
CMI	2.65	2.89	−1.363	0.173
RW value			44.929	<0.001
RW < 1.5	2669 (76.04)	4630 (74.58)		
RW 1.5–5	383 (10.91)	527 (8.49)		
RW 5–10	294 (8.38)	757 (12.19)		
RW > 10	164 (4.67)	294 (4.74)		
Number of organ transplant surgeries, n (%)	497 (14.16)	1134 (18.27)	27.082	<0.001
LT	196 (39.44)	360 (31.75)	9.096	0.003
RT	295 (59.36)	763 (67.28)	9.530	0.002
LT&RT	3 (0.60)	6 (0.53)	0.035	0.852
Other organs	3 (0.60)	5 (0.44)	0.187	0.665
*Mean postoperative hospital stay (day)*				
LT	30.17	26.73	−2.758	0.006
RT	25.23	23.31	−1.682	0.092
TEI	0.83	0.83	−0.352	0.725
CEI	1.40	1.21	−3.794	<0.001
IMLRG rate	0.00	0.00	—	—

DRGs, diagnosis-related groups; LT, liver transplant; RT, renal transplant; CMI, case-mix index; RW, risk weight; CEI, cost efficiency index; TEI, time efficiency index. IMLRG, inpatient mortality of low-risk group cases.

#### Distribution of transplant operations

The total number of pre-IFLT period and post-IFLT period transplant cases was 497 and 1,134, respectively. The proportion of pre-IFLT period and post-IFLT period transplant cases in the total number of discharged cases was 14.16 and 18.27%, respectively (*p* < 0.001). Meanwhile, the number of liver transplantation cases with pre-IFLT period and post-IFLT period accounted for 39.44 and 31.75% of total organ transplantation cases, respectively (*p* = 0.003). The number of kidney transplantation cases with pre-IFLT period and post-IFLT period accounted for 59.36 and 67.28% of total organ transplantation cases, respectively (*p* = 0.002). In addition, the number of combined liver and kidney transplantation cases with pre-IFLT period and post-IFLT period accounted for 0.60 and 0.53% of the total number of transplantation cases, respectively (*p* = 0.852).

#### Postoperative hospitalization days

Compared with the pre-IFLT period, the mean postoperative hospital stay was reduced by 11.40% (30.17 vs. 26.73 days) for liver transplant cases and 7.61% (25.23 vs. 23.31 days) for kidney transplant cases.

#### Distribution of major DRGs

**Figure 5:**
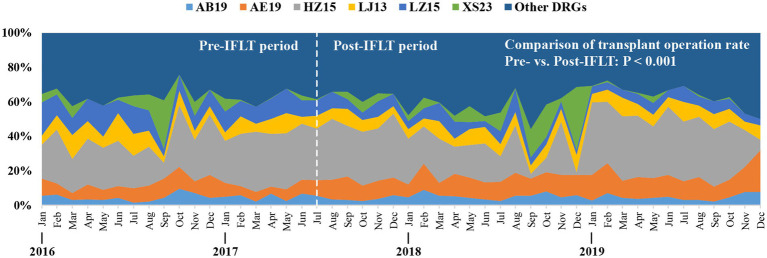
The ratio of major DRGs varies monthly from 2016 to 2019. DRGs, Diagnosis-related groups. Definition of main DRGs in the organ transplant department: AB19, Liver transplant. AE19. Renal transplant. HZ15, Other liver diseases without comorbidities and concomitant diseases. LZ15, Other urinary system diseases, without comorbidities and concomitant diseases. LJ13, other operations of the urinary system with comorbidities and concomitant diseases. XS23, followed up patients with complications and concomitant diseases.

As shown in Figure 5, there were six DRG groups associated with the majority of cases throughout the study period. Specifically, the six DRGs are AB19 (Liver transplant), AE19 (Renal transplant), HZ15 (Other liver diseases without comorbidities and concomitant diseases), LZ15 (Other urinary system diseases, without comorbidities and concomitant diseases), LJ13 (other operations of the urinary system with comorbidities and concomitant diseases) and XS23 (followed up patients with complications and concomitant diseases). The total number of cases in the above six DRGs accounts for more than half of the total number of cases.

### Efficiency of medical services

As shown in Figure 4B, TEI fluctuated around 0.83 throughout the study period, and there is no significant difference between TEIs in pre-IFLT and pre-IFLT periods (*p* = 0.725). At the same time, CEI showed a downward trend, and there was a significant statistical difference between the pre-IFLT period and the pre-IFLT period (*p* < 0.001, Figure 4C).

### Safety of medical services

In this study, the low-risk group was the DRG group admitted to the organ transplantation department with a low risk of death. As shown in Table 4, the mortality rate of low-risk cases in the organ transplant department was 0.00 both in the pre- and post-IFLT periods. During the study period, the distribution of patients at different risk grades of death in the organ transplant department was analyzed. As shown in Table 5, compared with the pre-IFLT period, the proportion of low-risk cases admitted to the organ transplantation department in the post-IFLT period decreased, while the proportion of high-risk cases increased (*p* < 0.001), suggesting that more high-risk cases were admitted to the department after the application of IFLT technology.

**Table 5:** Distribution of patients with different risk grades of death admitted to the organ transplant department in the pre- and post-IFLT periods.

Mortality risk grade	Pre-IFLT	Post-IFLT	*χ^2^* value	*p* value
	*n* (%)	*n* (%)		
No risk	247 (7.04)	405 (6.52)	119.738	<0.001
Low risk	877 (24.98)	1096 (17.66)		
Low-middle risk	1280 (36.47)	2878 (46.36)		
Middle-high risk	750 (21.37)	1166 (18.78)		
High risk	356 (10.14)	663 (10.68)		

LT, liver transplant; RT, renal transplant; IFLT, ischemia-free liver transplant.

The annual number of transplant operations performed by the organ transplant department during the study period was analyzed. As shown in Table 6, the total number of organ transplantation cases increased year by year from 2016 to 2019. Specifically, the annual number of implementation cases of CLT fluctuates between 110 and 130; The annual number of IFLT implementation cases fluctuates between 15 and 20; The annual number of RT cases has increased from 203 in 2016 to 353 in 2019.

**Table 6:** Annual number of transplants performed in the department during the study period.

**Number of organ transplant surgeries n (%)**	**2016**	**2017**	**2018**	**2019**
CLT	124 (37.35)	131 (34.47)	131 (30.61)	116 (23.63)
IFLT	0 (0.00)	16 (4.21)	19 (4.44)	19 (3.87)
RT	203 (61.15)	232 (61.05)	270 (63.08)	353 (71.89)
LT&RT	2 (0.60)	1 (0.26)	5 (1.17)	1 (0.20)
Other organs	3 (0.90)	0 (0.00)	3 (0.70)	2 (0.41)
Total	332 (100.0)	380 (100.0)	428 (100.0)	491 (100.0)

LT, liver transplant; RT, renal transplant; IFLT, ischemia-free liver transplant.

In addition, the composition of the top five DRGs groups in the low- and high-risk groups admitted to the organ transplant department during the pre-IFLT and post-IFLT periods was explored, respectively. As shown in Supplementary Table S1, the top three patients in the low-risk group admitted to the department were LZ15, LJ13 and LJ15, while the top three patients in the high-risk group admitted to the department were AB19, HR15 and HJ13 both in the pre-IFLT and post-IFLT periods.

### Ischemia-free transplant improves postoperative recovery

**Figure 6:**
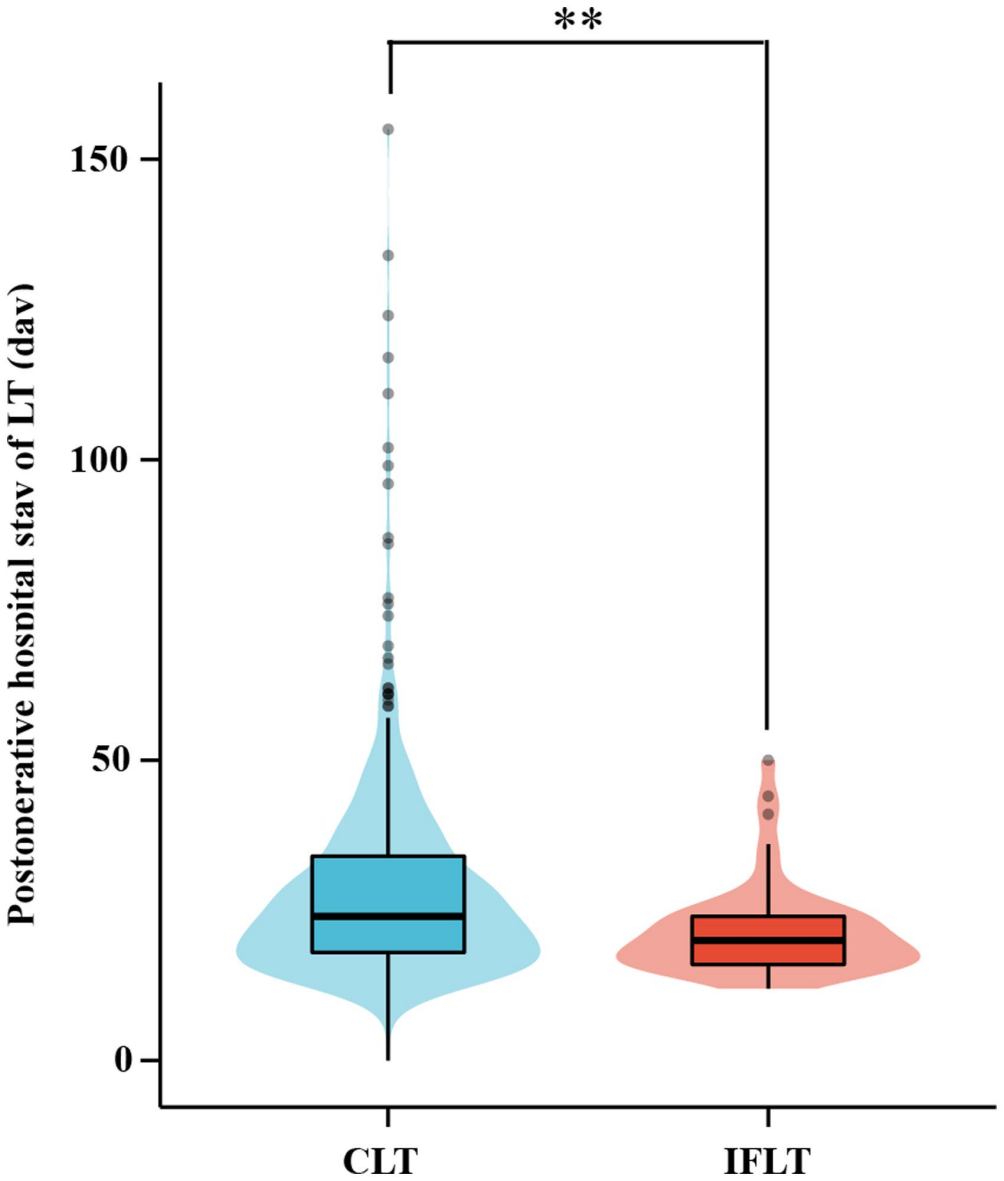
Comparison of postoperative hospital stay between CLT and IFLT in 2016 ~ 2019. CLT, conventional liver transplant. IFLT, ischemia-free liver transplant. LT, liver transplant. ^*^p < 0.05. ^**^p < 0.01. ^***^p < 0.001.

The length of hospital stay after surgical treatment reflects the recovery of patients after surgery. Therefore, the mean length of postoperative hospital stay was compared between patients with CLT and those with IFLT. As shown in Figure 6, the average length of postoperative hospital stay in patients with IFLT was significantly shorter than that in patients with CLT, suggesting that patients with IFLT had better postoperative recovery than those with CLT (*p* = 0.001).

## Discussion

Ischemia-free transplantation technology can effectively improve the clinical outcome of organ transplant (26). In order to assess the impact of this innovative technology on the medical service performance, we used the DRGs tool to evaluate the medical service performance in the organ transplant department of Sun Yat-sen university’s first hospital from 2016 to 2019. The results showed that after the IFLT implementation, the medical service performance indicators of organ transplant department showed an improvement trend, suggesting that the IFLT implementation of improved the medical service capacity of the department.

Normal machine perfusion (NMP) is a method of organ preservation that protects donor organs between acquisition and transplantation (27), contributes to organ utilization and improves post-transplant outcomes (28, 29). Previous studies have shown that the application of NMP can prevent the occurrence of IRI by inhibiting inflammation and promoting graft regeneration (30). However, as we know, the combination of NRP and HMP is often used for machine perfusion in the clinical practice of organ transplant (31). In this case, IRI may still occur in donor organs. Therefore, the hospital where this study was conducted successfully carried out the first IFLT in the world. IFLT enables the donor organ to obtain blood supply and support throughout the whole process, avoiding the IRI of the donor organ and reducing the risk of delayed graft function recover and acute rejection after surgery, thus further improving the transplant effect (32). The clinical practice shows that this innovative technology plays a positive role in promoting the medical service performance of the organ transplant department.

The payment method based on the DRGs case mix is a promising way for medical charging services (33, 34). It is reported that DRGs can help shorten hospital stay (35), reduce operations requiring expensive surgical instruments (36), and reduce medical costs (37). Although a lot of basic work needs to be carried out to realize DRG, including establishing adequate infrastructure, improving human resource capacity and improving the information management system (38, 39), these works are of great value, because scientific evaluation of medical service performance helps to improve clinical practice. At present, the DRGs tool has been widely concerned and applied to several clinical professional medical service evaluation (40). As far as we know, there is no research on the medical performance evaluation of organ transplant specialty using DRGs. Our research showed that DRG was an effective tool to evaluate the medical service performance of organ transplant specialty.

In this study, we used the CN-DRGs tool to analyze the medical service performance of organ transplant cases in the hospital. The results showed that the medical service performance in the organ transplant department showed an upward trend after the IFLT implementation. Specifically, in terms of medical income, the income structure of organ transplant department was more optimized. In terms of medical service capacity, both the number of DRGs and CMI showed an upward trend. The proportion of cases with higher RW (such as RW 5–10) increased, indicating that the type, scope and average technical difficulty of cases treated by organ transplantation department improved. It is worth mentioning that the proportion of organ transplant cases increased significantly during the post-IFLT period, indicating that the performance of professional medical service in this department improved. In terms of service efficiency, there was no statistical difference of TEIs between pre- and post-IFLT implementation, suggesting that the organ transplant department should pay more attention to the LOS in the future clinical practice. Meanwhile, the difference of CEIs pre- and post-IFLT was statistically significant, indicating that the cost of treating the same disease in organ transplantation department was significantly reduced. In terms of service safety, the mortality rate of low-risk cases in the organ transplant department was 0 throughout the study period, reflecting that the service safety of the department was good. Therefore, the indicators of DRGs, including TW, DRGs, CMI, TEI and CEI, have improved to varying degrees after the application of IFLT, suggesting that IFLT can help improve the medical service performance of the organ transplantation department. It is worth mentioning that, as shown in Figure 4C, compared with the CEI in 2016, the CEI in 2019 was significantly reduced, suggesting significant optimization in terms of hospitalization cost indicators. This may be related to the fact that IFLT recipients recover better after surgery, resulting in lower costs. The above results suggest that the application of IFLT can improve the medical service performance of organ transplantation department. Meanwhile, DRGs tools can accurately assess medical service performance.

Moreover, compared with CLT cases, the average postoperative LOS decreased significantly, suggesting that IFLT can effectively improve the postoperative recovery of patients. Previous studies suggest that there is a difference in the LOS after LT between China and the United States. According to data released by the Scientific Registry of Transplant Recipients (SRTR, https://www.srtr.org/) on January 5, 2023, the median postoperative LOS after LT in the United States was 10 days. Meanwhile, the postoperative LOS after LT varies by medical institution in the United States. For example, the median postoperative LOS after LT at Mayo Clinic Hospital Arizona was 6 days, while median postoperative LOS after LT at UF Health Shands Hospital was 14 days. Compared with that in the United States, the median postoperative LOS after LT in China was relatively longer. For example, a retrospective study from Beijing suggested that the median postoperative LOS after LT was 16 days (41). In addition, a retrospective study from Shanghai suggested that the median postoperative LOS after pediatric living donor LT was 24 days (42). In the present study, the mean postoperative LOS after LT in the post-IFLT period was 26.73 days, which was similar to the data reported by other medical institutions in China.

We believe that the reasons why LT patients in China have longer postoperative LOS compared to that in the United States may include the following: Firstly, there are differences in the medical systems of the two countries. Medical institutions in China have different practices in terms of care and management after LT, which may require a longer hospital stay. Secondly, there are differences in the characteristics of patients. Chinese patients may have different risk factors, which may require more intensive in-hospital monitoring, thus extending the LOS. Thirdly, there is a difference in cultural and social factors. Chinese families are likely to become more involved in caring for their family members during hospital stays, which could extend the LOS. Finally, differences in alternative therapy or medication use may also affect LOS.

There were several potential limitations in this study. Firstly, medical service performance evaluation based on DRGs requires high-precision FPMR. FPMR data used in this work was provided by the medical record management department of the hospital studied. Although the disease and surgery coding in FPMR had been under standard quality control, there is still the possibility of inaccurate coding, which may affect the accuracy of the evaluation results. Secondly, there was a lack of localized DRGs suitable for Guangdong Province, which may affect the accuracy of medical service performance evaluation data.

In conclusion, this study showed that medical performance indicators including total weight, CMI, CEI, and TEI of patients admitted to the organ transplant department in the post-IFLT period were improved to varying degrees compared with those in the pre-IFLT period, suggesting that the application of IFLT technology could contribute to improving the medical service performance of the organ transplant department. Meanwhile, the DRGs tool may help transplant departments to coordinate the future delivery planning of medical service.

## Data availability statement

The original contributions presented in the study are included in the article/Supplementary material, further inquiries can be directed to the corresponding authors.

## Author contributions

WZ and WC designed the study and revised the manuscript. JL, ZL, and YX collected and analyzed the data. JL interpreted the data and drafted the manuscript. HP, YZ, ZX, YL, and ZS revised the manuscript. All authors contributed to the article and approved the submitted version.

## Conflict of interest

The authors declare that the research was conducted in the absence of any commercial or financial relationships that could be construed as a potential conflict of interest.

## Publisher’s note

All claims expressed in this article are solely those of the authors and do not necessarily represent those of their affiliated organizations, or those of the publisher, the editors and the reviewers. Any product that may be evaluated in this article, or claim that may be made by its manufacturer, is not guaranteed or endorsed by the publisher.
