# Supplementary material for: Performance assessment of medical service for organ transplant department based on diagnosis-related groups: A programme incorporating ischemia-free liver transplantation in China
Source: Front Public Health. 2023 Apr 6;11:1092182. doi: 10.3389/fpubh.2023.1092182 (PMC10116067; doi:10.3389/fpubh.2023.1092182)
Supplement: Supplementary file 1 [file Data_Sheet_1.docx]

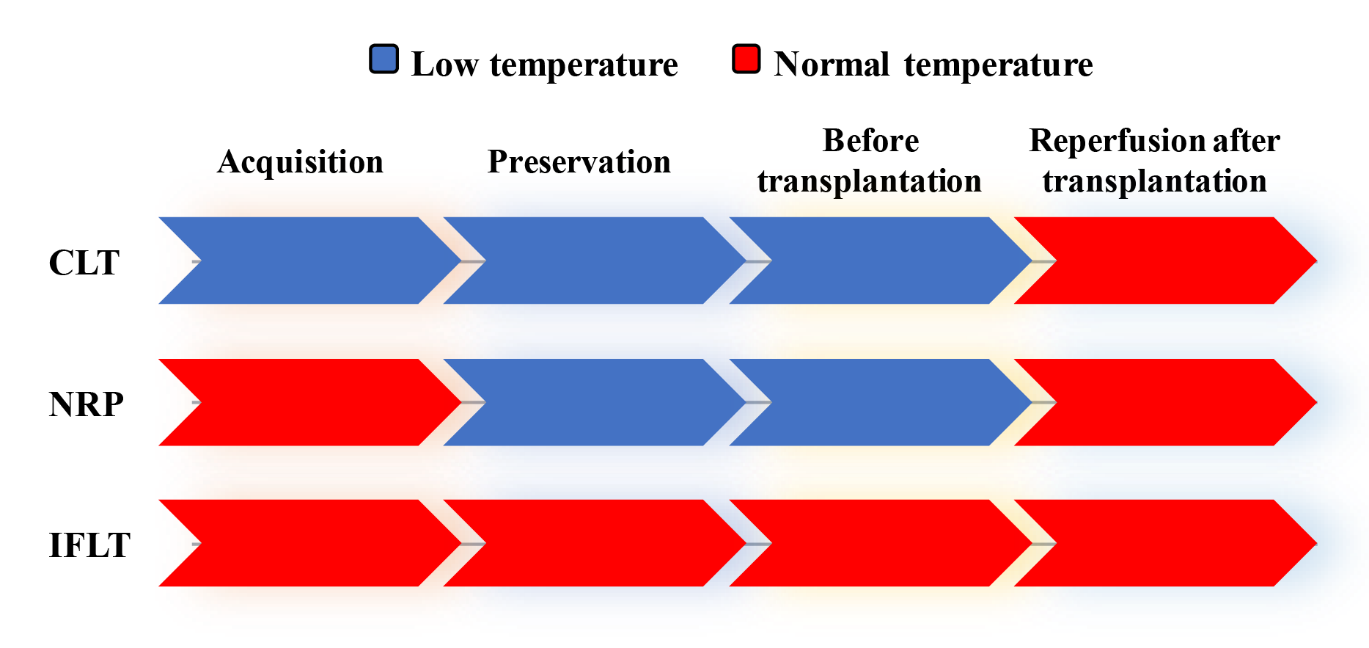


**Figure S1 Comparison of three application methods in liver transplant.** CLT, conventional liver transplant. NRP, normothermic regional perfusion. IFLT, ischemia-free liver transplant.

**Table S1 Top five DRGs in the low- and high-risk groups admitted to the organ transplantation department during the pre-IFLT and post-IFLT periods**

| **Rank** | **Pre-IFLT** | | **Post-IFLT** | |
| --- | --- | --- | --- | --- |
|  | **Low risk^a^** | **High risk^b^** | **Low risk** | **High risk** |
|  | DRG term, n (%) | DRG term, n (%) | DRG term, n (%) | DRG term, n (%) |
| 1 | LZ15, 402 (45.8) | AB19, 163 (45.8) | LZ15, 387 (35.3) | AB19, 290 (43.7) |
| 2 | LJ13, 291 (33.2) | HR15, 112 (31.5) | LJ13, 288 (26.3) | HR15, 206 (31.1) |
| 3 | LJ15, 79 (9.0) | HJ13, 40 (11.2) | LJ15, 121 (11.0) | HJ13, 60 (9.0) |
| 4 | GU15, 22 (2.5) | GJ13, 11 (3.1) | LB15, 118 (10.8) | HR11, 19 (2.9) |
| 5 | LV13, 21 (2.4) | HR11, 11 (3.1) | RU39, 57 (5.2) | HV11, 16 (2.4) |
| Other DRGs | 17 groups, 62 (7.1) | 8 groups, 19 (5.3) | 25 groups, 125 (11.4) | 14 groups, 72 (10.9) |

***a****. Low risk DRG terms above include LZ15- other diseases of urinary system, no comorbidities and comorbidities; LJ13- Other operations of urinary system with comorbidities and comorbidities; LJ15- Other operations of the urinary system, without complications or comorbidities; GU15- Esophagitis, gastroenteritis, without complication or concomitant disease; LV13- Urinary symptoms and signs with comorbidities and comorbidities; LB15- Kidney, ureteral, bladder surgery except major surgery for malignant tumors, without complications or concomitant diseases; RU39- Immunotherapy and/or other treatments for malignant proliferative diseases, etc.* ***b****. High risk DRG terms above include AB19- Liver transplantation; HR15- Malignant tumors of the hepatobiliary and pancreatic system without complications or concomitant diseases; HJ13- Other procedures related to liver, gallbladder, or pancreatic diseases with significant comorbidities and comorbidities; GJ13- Other operations of the digestive system with comorbidities and comorbidities; HR11- hepatobiliary pancreatic system malignancy with important comorbidities and comorbidities; HV11- Cirrhosis and alcoholic hepatitis with comorbidity and comorbidity, etc. LT, liver transplant; RT, renal transplant; IFLT, ischemia-free liver transplant.*
